# Biochemical Stability and Microbial Control of Reconstituted DaxibotulinumtoxinA-lanm for Injection

**DOI:** 10.3390/toxins15120683

**Published:** 2023-12-05

**Authors:** Kimberlee Ellis, Thai Thach, Conor J. Gallagher

**Affiliations:** Revance Therapeutics, Inc., Newark, CA 94560, USA

**Keywords:** botulinum toxin, DAXI, daxibotulinumtoxinA, drug stability, drug storage, neurotoxins, proliferation, microbial growth

## Abstract

DaxibotulinumtoxinA-lanm for injection (DAXI) is a unique US Food and Drug Administration-approved product comprising daxibotulinumtoxinA and a stabilizing excipient peptide (RTP004). DAXI has a longer-labeled shelf life (72 h) following reconstitution than other botulinum toxin type A products. Here, we report the stability and microbial control of reconstituted DAXI when stored at 2 °C–8 °C over a period of 36 days (Study 1) and 7 days (Study 2) following reconstitution with unpreserved or preserved saline. The pH and biological activity of reconstituted DAXI in the 50 U/vial and 100 U/vial formats remained stable at the final assessed time point in both preserved and unpreserved saline when refrigerated (2 °C–8 °C). No changes in recoverable 150 kDa neurotoxin (measured by enzyme-linked immunosorbent assay) were observed over 6 days of refrigeration. Bacterial growth or pathogen proliferation was not observed in DAXI reconstituted in preserved or unpreserved saline in both studies.

## 1. Introduction

Botulinum toxin type A (BoNTA) is a neurotoxin that disrupts cholinergic communication at the neuromuscular junction. BoNTA products are used worldwide for therapeutic and aesthetic indications, such as limb spasticity, focal dystonia, and facial wrinkles [1]. Commercially available BoNTA products vary in terms of the quaternary structure of the neurotoxin, the formulation excipients, quantity of core neurotoxin, and storage and handling recommendations [2]. All BoNTA products available in the US are dried in the manufacturing process and require reconstitution with saline prior to injection. Once reconstituted, manufacturers recommend refrigerated storage of BoNTA products at 2 °C–8 °C and use within 4 to 24 h, depending on the product [1,3]. As with any product intended for injection, storage beyond recommended guidelines is not recommended because of the risks of microbial contamination and reduced efficacy. Storage recommendations are based on stability and microbial growth studies that are conducted as part of the product development process, prior to marketing authorization. These data are required by regulatory agencies to provide guidance to practitioners on recommended handling and storage of the product. Alongside clinical and toxicological data, stability studies provide reassurance that storage under approved conditions guarantees the quality of the product for a limited time and post-reconstitution. These findings allow clinicians to make informed decisions about storage conditions post-reconstitution, which have implications for real-world clinical practice.

In clinical trials for BoNTA product approvals, the products are reconstituted in normal (unpreserved) saline (0.9% NaCl) as reflected in the product labeling [1,3]. However, in clinical practice, it is common for injectors to reconstitute botulinum toxins in preserved saline (i.e., bacteriostatic saline: 0.9% NaCl preserved with benzyl alcohol) [4], as it has been found that this results in less procedural pain than when BoNTA is reconstituted in unpreserved saline [5,6,7,8,9]. The product’s stability and sterility after reconstitution are influenced by the excipients formulated with the active pharmaceutical ingredient, in addition to the diluent. Traditional BoNTA products are formulated with differing amounts of human serum albumin (HSA) as a stabilizer and frequently include a sugar as a lyoprotectant [1]. However, these BoNTA formulations have a relatively short shelf life after reconstitution (4–24 h) [1,2,3] and, per the prescribing instructions, any product not administered to a patient within the indicated time frame should be considered unusable.

DaxibotulinumtoxinA-lanm for injection (DAXI; DAXXIFY^®^, Revance Therapeutics, Inc., Nashville, TN, USA), which is available in two vial formats (a 50 U/vial and a 100 U/vial) is a unique US Food and Drug Administration (FDA)-approved BoNTA formulation with different excipients and a longer-labeled shelf life of 72 h after reconstitution [10]. Rather than using HSA as a stabilizer, DAXI uses a proprietary 35-amino acid peptide (RTP004) as an excipient, which limits toxin aggregation and helps to prevent adsorption of BoNTA molecules to container surfaces. The primary objective of this study was to characterize the stability and sterility of reconstituted DAXI when stored at 2 °C–8 °C following reconstitution beyond the recommended storage time, using unpreserved saline and preserved saline to mimic clinical practice. A secondary objective was to confirm the stability and sterility of the 100 U/vial following reconstitution with unpreserved saline.

## 2. Results

### 2.1. Stability

**Figure 1:**
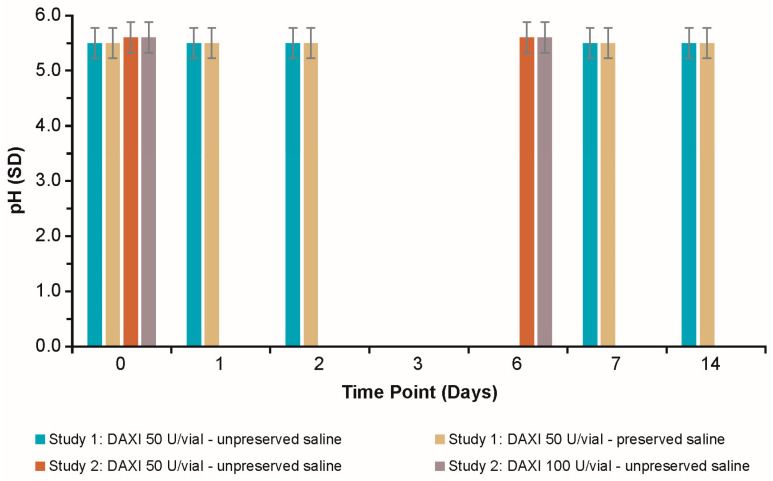
pH of reconstituted DAXI after 0–14 days of storage at 2 °C–8 °C. DAXI = DaxibotulinumtoxinA-lanm for injection; SD = standard deviation.

The pH of reconstituted DAXI remained stable at 5.5−5.6 at all time points in Study 1 and Study 2 (up to 14 days at 2 °C–8 °C; Figure 1). The pH remained stable when DAXI was reconstituted in both unpreserved and preserved saline for the 50 U/vial, and in unpreserved saline for the 100 U/vial, with no significant differences (*p* ≥ 0.05) between groups. 

**Figure 2:**
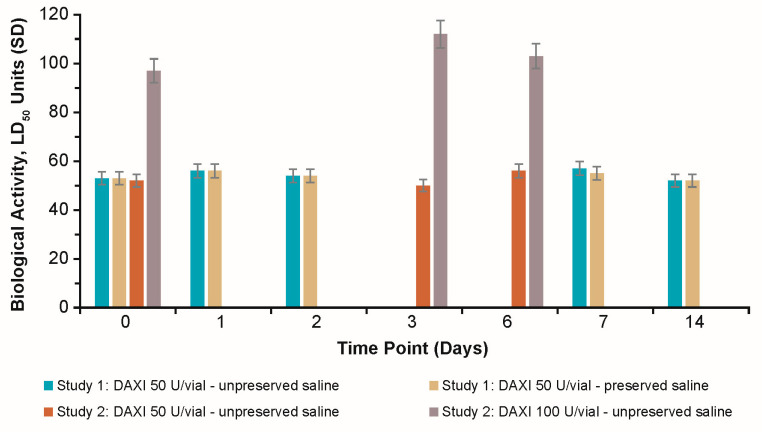
Potency of reconstituted DAXI after 0–14 days of storage at 2 °C–8 °C. DAXI = DaxibotulinumtoxinA-lanm for injection; LD_50_ = median lethal dose; SD = standard deviation.

The potency of reconstituted DAXI, as measured by mouse median lethal dose (LD_50_), was unchanged after refrigerated storage for 14 days in Study 1, and at the final time point of 6 days in Study 2 (Figure 2). The biological activity of reconstituted DAXI in the 50 U/vial remained constant and was similar over time in unpreserved and preserved saline, and remained constant over time in the 100 U/vial in unpreserved saline. 

The amount of 150 kDa neurotoxin (as measured by enzyme-linked immunosorbent assay [ELISA]) present within reconstituted DAXI remained stable over 6 days of refrigeration (*p* ≥ 0.05 between groups). In the 50 U/vial, the percentage change from baseline was +15% at day 3 and +5% at day 6 (coefficient of variation (CV): 7.3%), while in the 100 U/vial, the percentage change was −7% and −9%, respectively (CV: 5.1%). As biological activity as measured by mouse LD_50_ was preserved in both the 50 U and 100 U vials, the variability in recoverable neurotoxin is likely a function of the ELISA assay, rather than an actual decline in neurotoxin.

### 2.2. Microbial Control and Pathogen Proliferation

**Figure 3:**
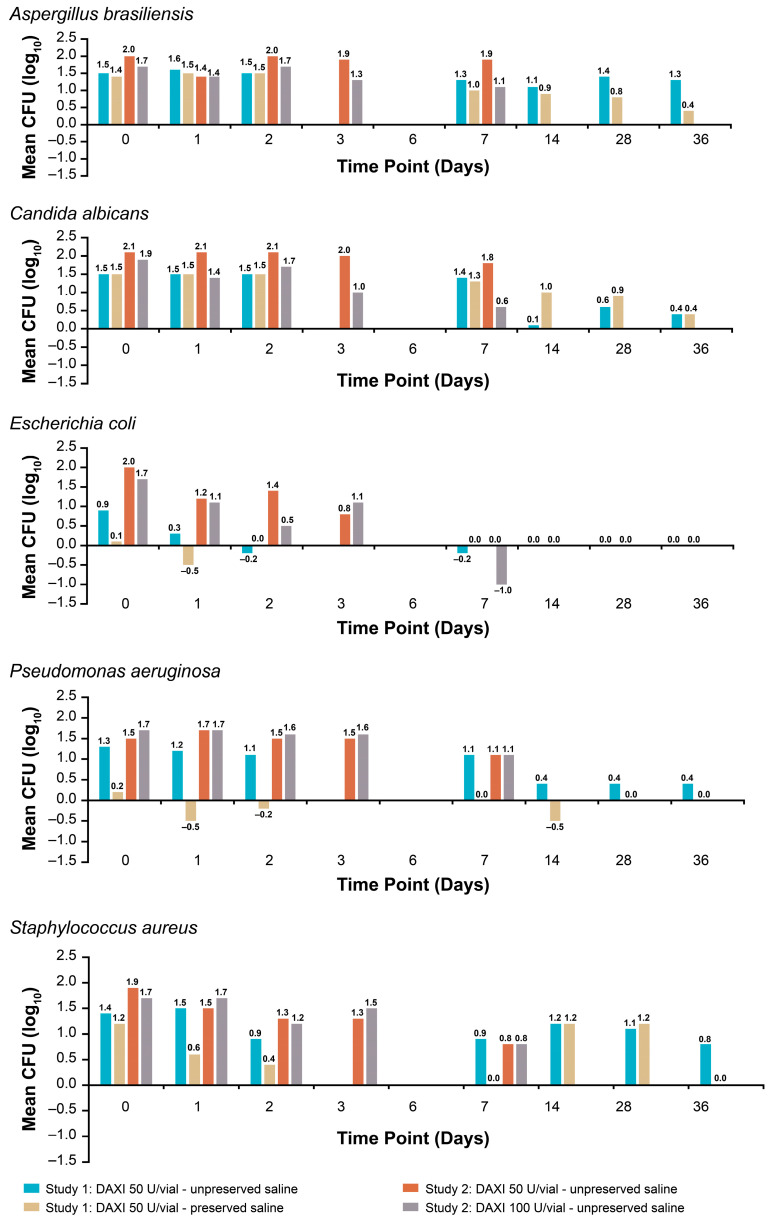
Pathogen proliferation in reconstituted DAXI after 0–36 days of storage at 2 °C–8 °C. CFU = colony-forming units; DAXI = DaxibotulinumtoxinA-lanm for injection.

No detectable bacterial growth was observed over 6 days of storage under refrigeration in either the 50 U/vial or 100 U/vial formats in unpreserved saline (colony-forming units [CFU]/mL < 1; Table 1). Furthermore, proliferation of the challenge pathogens was not observed in reconstituted DAXI over 36 days (Study 1) or 7 days (Study 2) at 2 °C–8 °C (Figure 3). No pathogen proliferation was observed when DAXI was reconstituted in preserved or unpreserved saline in the 50 U/vial or in unpreserved saline in the 100 U/vial.

## 3. Discussion

As BoNTA products are reconstituted before use, the characterization of stability and sterility over extended periods forms an important part of regulatory submissions, as well as being relevant information to inform clinical practice. DAXI reconstituted in the 50 U/vial, in either unpreserved or preserved saline, was biochemically stable in refrigerated (2 °C–8 °C) storage for up to 14 days. No changes in pH stability or recoverable 150 kDa neurotoxin content were observed, and in vivo biological activity was maintained. Reconstituted DAXI did not support microbial growth, as evidenced by an absence of bioburden and lack of pathogen proliferation over the duration of both studies. No differences in stability or microbial growth were observed between the 50 U/vial and 100 U/vial formats.

Legacy formulations of BoNTA containing HSA have a recommended storage time of no longer than 4 to 24 h at 2 °C–8 °C, following reconstitution of the product [1,3]. These time frames have been described as “excessively strict” and may lead to waste [3], and have led practitioners to use reconstituted BoNTA formulations that have been refrigerated for longer than the manufacturer-recommended time frame [11]. The formulation of DAXI differs markedly from other BoNTA products due to the inclusion of the peptide excipient RTP004, rather than HSA, which is the usual stabilizer used in BoNTAs. This unique formulation may contribute to increased stability following reconstitution, by preventing neurotoxin aggregation, and adsorption to the storage container. The stability of reconstituted DAXI substantially exceeds that of legacy formulations, with reconstituted DAXI remaining biochemically stable in both toxin content and biological activity at the final tested time point of 14 days of refrigerated storage. Based on the submitted data, the US FDA approved DAXI with labeling that indicated that a reconstituted vial could be held for 72 h post-reconstitution at 2 °C–8 °C.

We investigated the stability of DAXI when reconstituted in either preserved or unpreserved saline in the context of practitioners opting to follow off-label consensus recommendations and use preserved saline, which is known to reduce pain on administration [4,5]. DAXI’s formulation offers enhancements in the form of stability and the flexibility to reconstitute in either unpreserved or preserved saline. In clinical practice, the ability to store DAXI for longer periods than other BoNTA products may result in reduced wastage. For example, products reconstituted on a Friday may be safely used the following week.

Although microbial contamination is a potential issue with longer-term refrigerated storage of an agent, there was no evidence of bioburden in reconstituted DAXI after 6 days of refrigerated storage. In fact, no proliferation of any of the five challenge pathogenic organisms occurred in reconstituted DAXI in the 50 U/vial for up to 36 days or in the 100 U/vial for up to 7 days. 

This study provides information on the stability and microbial control of reconstituted DAXI in terms of duration of storage and use of preserved saline. However, in this investigation we did not evaluate other real-world conditions such as temperature excursions, exposure to light, and other variations in reconstitution practices that may affect product stability and sterility. These studies, which are not regulatory requirements, may be considered in the future.

## 4. Conclusions

DAXI is a uniquely formulated BoNTA agent with a longer duration of clinical effect than other BoNTA agents [12,13,14,15]. Reconstituted DAXI demonstrated excellent biochemical stability and microbial control when stored at 2 °C–8 °C in either preserved or unpreserved saline, without loss of activity or compromising sterility. These studies indicate that DAXI’s stability extends beyond the FDA-approved storage time of 72 h. While storage of reconstituted DAXI for longer than or outside the conditions stipulated on the product label is discouraged, the information presented here allows clinicians to evaluate the benefit/risk of storage and reconstitution practices in the real world.

## 5. Materials and Methods

### 5.1. Study Design

The biochemical stability and microbial control of DAXI after reconstitution and refrigerated storage at 2 °C–8 °C were evaluated in two discrete studies.

#### 5.1.1. Study 1

Study 1 investigated DAXI in the 50 U/vial format, reconstituted in 0.63 mL of unpreserved or preserved saline (0.9% NaCl or 0.9% NaCl with 0.9% benzyl alcohol). Stability was assessed by pH, and biological activity by mouse LD_50_ assay was evaluated over 14 days. Samples were prepared in duplicate and were analyzed on days 0, 1, 2, 7, and 14. Pathogen proliferation was assessed over 36 days at 2 °C–8 °C by challenge with five common pathogenic organisms (*Aspergillus brasiliensis, Candida albicans, Escherichia coli, Pseudomonas aeruginosa*, and *Staphylococcus aureus)* at ~100 CFUs each. Samples were prepared in duplicate and analyzed on days 0, 1, 2, 7, 14, 28, and 36.

#### 5.1.2. Study 2

Study 2 investigated DAXI in the 100 U/vial format after reconstitution in 1.2 mL of unpreserved saline using the 50 U/vial format, reconstituted in 0.63 mL of unpreserved saline, as a comparator. Stability was assessed by pH, analysis of biological activity, and determination of the amount of active pharmaceutical ingredient (150 kDA core neurotoxin) in ng/mL by ELISA over 6 days, with measurements on days 0, 3, and 6 (pH measured on days 0 and 6 only). Assessment of the maintenance of biological activity by LD_50_ assay was conducted on days 0, 3, and 6. Microbial control was measured by bioburden (over 6 days) and pathogen proliferation by challenge with the same five common pathogenic organisms described above at ~100 CFUs each. Samples were prepared in duplicate and analyzed over 7 days.

### 5.2. Stability Assays

#### 5.2.1. pH Measurement

The pH of the reconstituted solution was measured in accordance with USP <791> and Ph. Eur. 2.2.3. pH meters were standardized with pH solutions traceable to the National Institute of Standards and Technology. All measurements were performed using a calibrated pH meter at temperatures between 20 °C and 25 °C.

#### 5.2.2. Mouse LD_50_ Assay

All studies were executed in accordance with the procedures described in an approved protocol. Mouse studies were approved by the Institutional Animal Care and Use Committee: LifeSource Biomedical Services, LLC Institutional Animal Care & Use Committee (#REV-09-001-Y8, approved on 24th May 2017). Approval was granted in compliance with the Animal Welfare Act as well as standards described in the *Guide for the Care and Use of Agricultural Animals in Research and Teaching (The Guide)* and the *AVMA Guidelines for the Euthanasia of Animals: 2020 Edition*.

Female CD-1 mice at approximately 4 weeks old and weighing 17–23 g were used. Mice were administered a range of DAXI doses in saline via intraperitoneal injection. A total of seven dosing concentrations were used, with four dosing solutions per sample (two reference standards and two test samples) and eight mice per group. Lethality was recorded at 24, 48 (±4), and 72 (±2) h. LD_50_ data were analyzed by a constrained 7-dose, 4-parameter Probit curve analysis with a reference and test sample to determine biological activity of DAXI. 

### 5.3. ELISA

The stability of the 150 kDa neurotoxin was assessed using ELISA. A standard ELISA protocol was followed using a polyclonal, biotin-conjugated, rabbit anti-BoNTA IgG capture antibody, 1% bovine serum albumin and 9% casein in phosphate-buffered saline as a blocking agent, and streptavidin-conjugated horseradish peroxidase (Ultra Streptavidin-HRP, Thermo Fisher Scientific, Waltham, MA, USA) with 3,3′,5,5′-tetramethylbenzidine (^®^B Liquid Substrate System for ELISA, Sigma Aldrich, St. Louis, MO, USA) as the colorimetric detection reagents. Absorbance was read at 450 nm using a Wallace EnVision Multi-Label Microplate Reader (Perkin Elmer, Waltham, MA, USA). DAXI concentrations were calculated using a 4-parameter non-linear regression analysis. Accounting for the variability of the assay, the threshold for change in recoverable neurotoxin was <20%.

### 5.4. Microbial Control Assay

A microbiologic examination for the quantitative enumeration of bacteria and fungi was conducted using a membrane filtration technique in accordance with USP <61> and Ph. Eur. 2.6.12 for total microbial count and total yeast and mold. Reconstituted vials were filtered and rinsed through a 0.45 μm filter using a manifold filtration device. Filters with retained cells were separately incubated at 30 °C–35 °C on a trypticase soy agar (TSA) plate for 3–5 days to allow aerobic organisms to grow and at 20 °C–25 °C on a sabouraud dextrose agar (SDA) plate for 5–7 days. The procedure allows for the enumeration of CFUs, if present.

### 5.5. Pathogen Proliferation Assays

Five common pathogenic organisms were used as challenge organisms to determine whether reconstituted DAXI supported microbial growth: *A. brasiliensis, C. albicans, E. coli, P. aeruginosa*, and *S. aureus* (US Pharmacopeia, Rockville, MD, USA for Study 1). The assay was conducted in accordance with USP <61> and Ph. Eur. 2.6.12. Vials of reconstituted DAXI were spiked with ≤100 CFUs of the challenge organism and were stored for 0, 1, 2, 7, 14, 28, and 36 days (Study 1) or for 0, 1, 2, 3, and 7 days (Study 2), at 2 °C–8 °C, before plating onto TSA or SDA, depending on the challenge organism. CFUs were counted and the results were reported as log_10_ values. Samples for each condition were prepared in triplicate. Controls included a positive control (spiked saline without DAXI), air control, and negative control.

### 5.6. Statistical Analysis

Descriptive statistics are presented. Microbial data were analyzed using log reduction. Stability of the 150 kDa neurotoxin by ELISA is reported as the percentage change from baseline in neurotoxin content of reconstituted DAXI (ng/mL) after storage at 2 °C–8 °C. When reported, differences between groups were assessed using a two-sided t-test with an alpha of 0.05.

## Figures and Tables

**Table 1:** Bioburden in DAXI reconstituted in unpreserved saline after 0 or 6 days of storage at 2 °C–8 °C.

	Bioburden, CFU/mL	
Time Point, Days	DAXI 50 U/Vial	DAXI 100 U/Vial
0	<1	<1
6	<1	<1

n = 1. CFU = colony-forming unit; DAXI = DaxibotulinumtoxinA-lanm for injection.

## Data Availability

Data are contained within the article.
